# Effect of parecoxib on postoperative anxiety and cognitive function after pancreaticoduodenectomy: A clinical trial

**DOI:** 10.1097/MD.0000000000048330

**Published:** 2026-04-17

**Authors:** Fuzhen Zhang, Liping Ma, Xiaoyun Li, Shizhao Wang, Lichao Di

**Affiliations:** a Department of Anesthesiology, The Second Hospital of Hebei Medical University, Shijiazhuang, Hebei, China.

**Keywords:** cognitive function, pancreaticoduodenectomy, parecoxib, perioperative neurocognitive disorders, postoperative anxiety

## Abstract

**Background:**

After pancreaticoduodenectomy, perioperative neurocognitive disorders (PND) are a common complication. Parecoxib, a selective cyclooxygenase-2 inhibitor, is used for its analgesic and antiinflammatory effects, but its impact on postoperative anxiety and cognitive function is not clear.

**Methods:**

The patients undergoing laparoscopic pancreaticoduodenectomy at the Second Hospital of Hebei Medical University were enrolled in the study from November 1, 2020 to September 30, 2022 in this randomized controlled trial. Patients were randomly divided into Group P who received 20 mg of parecoxib intravenously 10 minutes before anesthesia and at the end of surgery and Group C who received an equal volume of normal saline at the same times. Primary outcomes included the effect of parecoxib on anxiety, cognitive impairment, and postoperative intestinal function recovery, with the incidence of PND calculated using the *Z*-value method. Secondary outcomes included pain control, length of hospital stay, postoperative recovery, complication rates, and analgesic use.

**Results:**

80 patients were enrolled and randomly divided into 2 groups of 40 equally: there were no significant differences in postoperative flatus passage, defecation time, hospital stay, or complication rates (*P* > .05). In terms of postoperative anxiety, the experimental group had significantly lower State Anxiety Inventory scores at 24 hours postoperatively (T2) (*P* < .05). Cognitive function, measured by Montreal Cognitive Assessment scores, did not show significant differences; however, cognitive decline was less pronounced in the experimental group. PND rates were 5% (2/40) in the experimental group versus 12.5% (5/40) in the control group, suggesting a possible clinical tendency toward reduced incidence, although the difference did not reach statistical significance. The rates of PND at 24 hours (T2) and 7 days (T3) postoperatively were 10% (4/40) and 5% (2/40) for the experimental group, compared to 15% (6/40) and 12.5% (5/40) for the control group using the *Z*-value method, Although the between-group differences did not reach statistical significance, a lower incidence was observed in the parecoxib group, suggesting a possible clinical tendency.

**Conclusion:**

Parecoxib, when used with pancreaticoduodenectomy, alleviates postoperative anxiety and neurocognitive disorders without affecting postoperative intestinal function recovery or increasing the incidence of postoperative complications.

## 1. Introduction

The typical surgical method for treating ampulla and periampulla tumors is laparoscopic pancreaticoduodenectomy (LPD). LPD is one of the most difficult surgical operations due to the large number of organs involved, the large surgical scope and the complicated reconstruction process of the digestive tract.^[1,2]^ While the operation causes trauma to the body, the perioperative recovery process also causes psychological stress damage to the patients.

Perioperative neurocognitive disorders (PND) refer to neurocognitive abnormalities detected during the perioperative periods.^[3]^ The pathogenesis includes neuroinflammation, cholinergic system dysfunction, stress, old age, anesthesia, surgery, and so on.^[4]^ Anxiety is a common subjective psychological feeling of patients after surgery, and the increase of anxiety after surgery exacerbates the generation of cognitive impairment to a certain extent.

Parecoxib is a novel nonsteroidal antiinflammatory drug, a selective inhibitor of cyclooxygenase-2 (COX-2), which has fewer side effects than traditional opioid analgesics and can be used for analgesic and antiinflammatory purposes in surgery.^[5]^ Currently, parecoxib is considered an optimal strategy for reducing opioid use after surgery according to the enhanced recovery after surgery guidelines. There are also studies showing that celecoxib, in addition to routine analgesic treatment, can improve postoperative cognitive function in patients.^[6]^ But few studies have explored the impact of parecoxib on the management of postoperative anxiety, cognitive impairment and postoperative intestinal function recovery.

This experiment is a prospective, randomized, and controlled clinical trial. The study evaluated preoperative and postoperative Montreal Cognitive Assessment (MoCA) and State-Trait Anxiety Inventory (STAI) scores to investigate how parecoxib affects postoperative anxiety and cognitive performance in individuals undergoing pancreaticoduodenectomy. Additionally, the effect of parecoxib on postoperative intestinal function was observed.

## 2. Materials and methods

### 2.1. Study design and patients

This study was approved by the Ethics Committee of he Second Hospital of Hebei Medical University. From November 1, 2020 to September 30, 2022, 80 patients undergoing LPD at the Second Hospital of Hebei Medical University were invited to participate in this randomized controlled clinical trial. Eighty participants were randomly divided into the intervention group (Group P) received parecoxib sodium and the control group (Group C) received the placebo using a computer-generated randomization sequence. The randomization sequence was created by an independent statistician who was not involved in the recruitment or treatment of participants. To ensure concealment of the allocation sequence, sequentially numbered, opaque, and sealed envelopes were used. Each envelope contained the group assignment for a participant.

The inclusion criteria included: patients undergoing elective LPD; aged 60 to 85 years; body mass index (BMI) between 18 and 30; classified as American Society of Anesthesiologists (ASA) Physical Status II to III, primary school education or above, and informed consent. Exclusion criteria included: those who have cognitive dysfunction or have a tendency to change their cognitive function in the past 1 year; people with a history of allergy to parecoxib for injection or other nonsteroidal antiinflammatory drugs (NSAIDs) or known hypersensitivity to sulfonamides; patients with active gastrointestinal ulcer or gastrointestinal bleeding; patients with congestive heart failure classified as NYHA class II to IV; patients with diagnosed ischemic heart disease, peripheral arterial, and/or cerebrovascular disease; patients with severe hepatic insufficiency defined as serum albumin levels below 25 g/L or a Child-Pugh score of at least 10; pregnant or nursing patients; patients with severe mental illness, taking sedatives or antidepressants, having alcohol or drug abuse, or using NSAIDs the day before surgery; people with difficulties in understanding and communicating.

The study was conducted in accordance with the Good Clinical Practice guidelines, Consolidated Standards of Reporting Trials criteria. All patients provided informed consent. This study was registered at the Chinese Clinical Trial Register (http://www.chictr.org.cn, ChiCTR1900024336, ChiCTR2000037746). It was approved by the Research Ethics Committee of the Second Hospital of Hebei Medical University (2019-P004, 2020-C049-F01).

### 2.2. Blinding

The following parties were blinded to the treatment allocation. Participants: participants were blinded to the treatment they received. They were not informed whether they were receiving the active drug (parecoxib) or the placebo. Care providers: the surgical and anesthetic teams providing care to the participants were also blinded to the treatment allocation. This ensured that any postoperative care was not influenced by knowledge of the treatment group. Outcome assessors: the personnel responsible for evaluating the primary and secondary outcomes (such as the MoCA and State Anxiety Inventory [SAI] scores) were blinded to the treatment allocation. This included the researchers conducting the assessments and the statisticians analyzing the data.

To achieve blinding, the following measures were taken. Drug preparation: the active drug (parecoxib) and the placebo were prepared to look identical in terms of appearance, packaging, and administration method. Only a designated pharmacist who was not involved in the care of the participants knew the actual contents of each dose. Randomization concealment: the randomization sequence was generated by an independent statistician who did not participate in the recruitment or treatment of the participants. The sequence was kept confidential and was only accessible to the pharmacist who prepared the drugs. Emergency unblinding: in case of an adverse event where knowledge of the treatment was necessary for the safety of the participant, an emergency unblinding procedure was in place. Each participant had an emergency envelope containing the information about the treatment allocation. This envelope would only be opened in emergencies by authorized personnel. Documentation: all efforts were made to maintain blinding throughout the trial. Any instances of unintentional unblinding were documented and reported in the final analysis. By implementing these procedures, we aimed to ensure that the assessment of outcomes was unbiased and that the results accurately reflected the effects of the intervention.

### 2.3. Intervention methods

Patients in Group P were given intravenous injection of parecoxib 20 mg 10 minutes prior to anesthesia and again at the end of surgery, while patients in group C were given an equal volume (5 mL) of normal saline at the same timepoints. Electrocardiogram, pulse oxygen saturation, arterial blood pressure, pharyngeal temperature, EEG bispectral index, and the pressure of end-tidal carbon dioxide were monitored continuously in all patients. Anesthesia was intiated using an intravenous injection of midazolam (0.05 mg/kg), cisatracurium (0.15 mg/kg), propofol (1.5 mg/kg), and sufentanil (0.3 μg/kg), followed by endotracheal intubation for subsequent mechanical ventilation. Maintenance of anesthesia was achieved through inhalation of 1% sevoflurane, continuous pump injection of propofol at a rate of 1 to 3 mg/(kg·h) and remifentanil at a rate of 0.1 to 0.3 μg/(kg·min) until the end of the operation, with intermittent intravenous injection of cisatracurium. Blood pressure was kept within a range of ±20% from the basic value, heart rate was maintained at 60 to 100 beats/min, pressure of end-tidal carbon dioxide was maintained at 35 to 45 mm Hg, and the value of EEG bispectral index was maintained in the range of 40 to 60. Dezocine 0.1 mg/kg was administered 30 minutes prior to the conclusion of the operation. After the operation, patient-controlled intravenous analgesia was used for all patients. The medication was a 100 mL mixture (sufentanil 1 μg/kg + dexmedetomidine 2 μg/kg + dezocine 0.4 mg/kg + normal saline). Patients were provided the option to self-administer 0.5 mL boluses with a 15-minute lockout interval, and the infusion rate was set at 2 mL/h. Opioids or tramadol were given intravenously as rescue analgesia when the pain feels unbearable.

### 2.4. Primary and secondary outcomes

The patient general status was recorded, including age, gender, BMI, ASA rating, years of education, as well as surgical information including surgical duration, blood loss, fluid rehydration, and intestinal status. The primary outcomes were anxiety levels and cognitive status before surgery (T1), 24 hours after surgery (T2), and 7 days after surgery (T3).

STAI is a tool for assessing anxiety levels in adults and includes the SAI and Trait Anxiety Inventory and used to assess patients’ anxiety levels at baseline and after surgery. The cognitive level at time points T1, T2, and T3 was evaluated using the MoCA scale, a widely-used screening tool designed to detect mild cognitive impairment. The total score is out of 30 points, with the following breakdown. Orientation: 6 points, assessing both temporal and spatial orientation, with 1 point awarded for each correct answer. Executive functions: 5 points, evaluated through tasks such as drawing a closed clock face with a specified time and performing a letter-number sequencing task. Memory: immediate recall of 5 words. Attention: 6 points, includes sustained attention tests (e.g., serial sevens), forward digit span, and visual scanning tasks. Language: 6 points, assessing verbal fluency (naming animals), sentence repetition, and object naming. Abstraction: 2 points, requiring the participant to identify common features between 2 provided concepts. Delayed recall: 5 points, recalling the previously mentioned 5 words at the end of the assessment, with 1 point per word remembered. A total score of 26 or above is generally considered indicative of normal cognitive function. Note that an additional point may be added to the total score for individuals with <12 years of education to account for educational disparities.^[7]^ The “*Z*-score method” recommended by the International Study of PostOperative Cognitive Dysfunction (ISPOCD) was used to evaluate whether the patients had cognitive impairment.^[8]^ When the *Z*-score of the patient was >1.96, the patient was considered to have PND. *Z*-value = (postoperative change of the patient from before − mean of the change of the control group)/standard deviation of the change of the control group.

The secondary outcomes included the recovery time of intestinal function, visual analogue scale (VAS) score for pain, duration of postoperative hospitalization, and postoperative adverse events such as nausea, vomiting, vein thrombosis, anastomotic fistula, incision infection, and the need for a 2nd operation due to bleeding. The recovery time of intestinal function was determined by the time taken for the patient to 1st exhaust (pass gas) and defecate after surgery. This measure reflects the return of bowel motility and digestive function.^[9]^ Pain intensity was assessed using the VAS, a widely accepted and validated instrument for measuring pain in clinical practice.^[10]^ The VAS score ranges from 0 to 10, where 0 indicates no pain and 10 indicates the worst imaginable pain. Scoring range: 0 (no pain) to 10 (worst imaginable pain). The duration of postoperative hospitalization was recorded as the number of days from the date of surgery until discharge. This outcome is an important indicator of resource utilization and patient recovery.^[11]^ Postoperative adverse events were evaluated, including nausea, vomiting, vein thrombosis, anastomotic fistula, incision infection, and the need for a 2nd operation due to bleeding. These complications were identified and documented according to standard clinical criteria.^[12]^

### 2.5. Sample size

The sample size for this study was calculated based on the primary outcome of interest, which was the change in SAI scores from baseline to 24 hours postsurgery. We aimed to detect a clinically meaningful difference in SAI scores between 2 group. We referred to previous studies^[13,14]^ that reported the standard deviation of SAI scores in similar populations. Based on these studies, we assumed a standard deviation of 5 points for SAI scores. A clinically significant difference of 8 points in SAI scores was considered.^[15]^ The sample size was calculated using G*Power 3.1 software (Heinrich-Heine-Universität Düsseldorf, Düsseldorf, Germany),^[16]^ with the following parameters: effect size (*d*): 1.6 (calculated as the difference in means divided by the pooled standard deviation); significance level (α): 0.05 (two-sided); power (1 − β): 0.80; tail(s): 2-tailed. Based on the preliminary study, a sample size of 86 patients (43 per group) was determined, anticipating a 10% dropout rate. Therefore, we aimed to recruit 43 participants per group, totaling 86 participants. Although 86 patients were initially planned for enrollment, 80 patients completed the study without loss to follow-up. No participants were excluded after randomization. The final sample size was slightly smaller than planned. This does not affect the statistical power for detecting small differences between groups.

### 2.6. Statistical analyses

Statistical analysis was performed using SPSS 20.0 (IBM Corp., Armonk). The Kolmogorov–Smirnov (*K–S*) test was employed for normality testing of the data in the 2 groups. For normally distributed continuous variables, they were presented as (mean ± standard deviation) and were analyzed using *t* tests. Nonnormally distributed continuous variables were represented as median (*Q*25, *Q*75) and were analyzed using the *U* test. Categorical data were presented as *n* (%) and were analyzed using Chi-square test. A significance level of *P* < .05 was considered to be statistically significant.

## 3. Results

### 3.1. Patient characteristics

**Figure 1.**
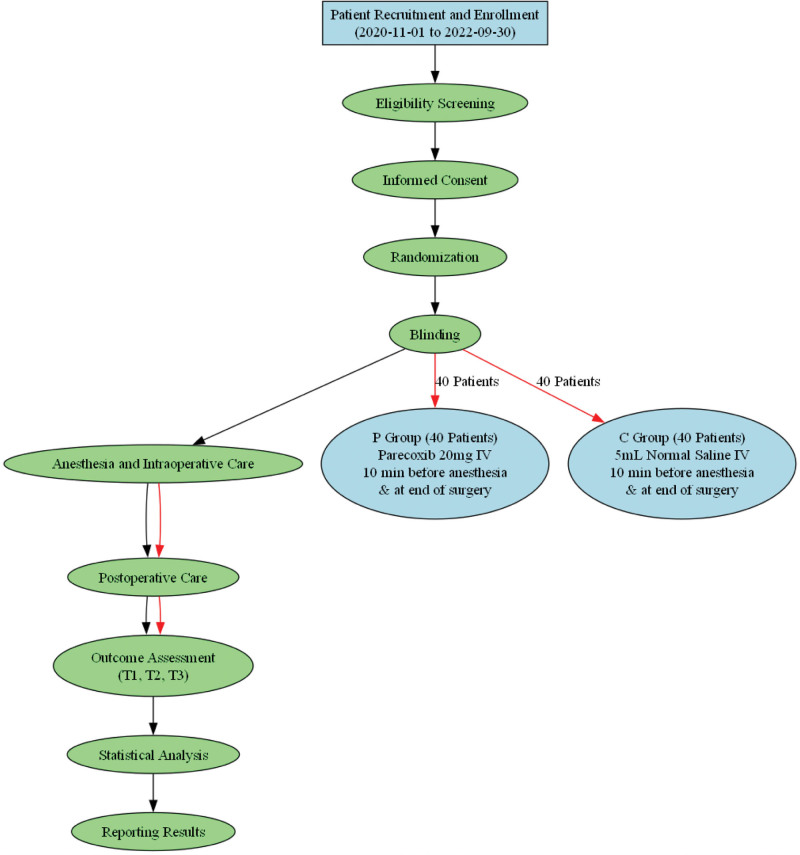
Study design and flowchart for the clinical trial assessing parecoxib in pancreaticoduodenectomy.

A total of 80 patients were randomized and all completed the study. No patients were lost to follow-up or excluded after randomization. 80 patients, including 40 from group P and 40 from group C, finished the trial in the end (Fig. 1). No statistically significant differences were observed between the 2 groups, including gender, age, BMI, years of education, ASA classification, operation duration, intraoperative blood loss, and fluid rehydration (Table 1).

**Table 1:** Comparison of general data and intraoperative conditions between the 2 groups.

	Group P (n = 40)	Group C (n = 40)	*T*/χ^2^	*P* value
Gender (M/F)	26/14	26/14	0.000	.999
Age	60.45 ± 9.93	62.39 ± 8.65	−0.932	.358
BMI	24.41 ± 2.79	23.50 ± 3.56	1.272	.210
ASA (I/II/III)	0/25/15	0/23/17	0.208	.648
Comorbidity (n)	21	29	3.414	.065
Normal defecation frequency	1.66 ± 1.20	1.43 ± 1.10	0.893	.357
Operation time (h)	5.85 ± 1.63	5.75 ± 1.28	0.305	.76
I.O. blood loss (mL)	420.50 ± 514.970	400 ± 480.92	0.184	.854
Fluid replenishment volume	3170.75 ± 834.88	3050.63 ± 777.21	0.666	.507
Abdominal distension (n)	10	11	0.0645	.799
Feeding capacity (n)	36	38	0.721	.396

I.O. = intraoperative, M/F = male/female, n = number.

### 3.2. Outcomes of surgery following surgical intervention

As shown in Table 2, there was no significant difference in the postoperative exhaust and defecation time, the incidence of nausea and vomiting, and postoperative hospitalization days between the 2 groups. In terms of postoperative complications, 1 case of intramuscular venous thrombosis occurred in group P, while 1 case of anastomotic fistula and 1 case of incision infection occurred in group C, showing no significant difference. There were no significant differences in the baseline Trait Anxiety Inventory and SAI scores between the 2 groups. However, at 24 hours postsurgery, the SAI scores of patients in Group P were significantly lower than those in Group C (*P* < .05). There was no statistically significant difference in cognitive function between the 2 groups, and the incidence of PND was 5% (2/40) in group P and 12.5% (5/40) in group C. There was no significant difference in VAS scores between the 2 groups.

**Table 2:** Outcomes of surgery following surgical intervention.

	Group P (n = 40)	Group C (n = 40)	*T*/χ^2^	*P* value
Exhausting time (h)	67.08 ± 34.39	63.25 ± 30.79	0.524	.602
Defecation time (h)	93.05 ± 35.74	101.38 ± 49.91	−0.858	.394
Postoperative hospital stay (d)	16.10 ± 8.82	15.50 ± 7.48	0.328	.744
Nausea (n)	11 (27.5%)	10 (25%)	0.0646	.799
Vomit (n)	4 (10%)	5 (12.5%)	0.125	.723
Postoperative adverse events (n)				
Intermuscular vein thrombosis	1 (2.5%)	0 (0%)	1.0126	.315
Incision seepage	0 (0%)	1 (2.5%)	1.0126	.315
Incision infection	0 (0%)	1 (2.5%)	1.0126	.315
Second operation (bleeding factor)	1 (2.5%)	1 (2.5%)	0.000	
Additional analgesia (n)	29	27	0.238	.626
Time to live bed (h)				
D1–D3	0	0	0.000	.999
D4	1.18 ± 2.63	1.78 ± 2.71	−1.005	.318
D5	3.88 ± 4.36	5.25 ± 5.71	−1.206	.230
STAI scores				
TAI	41.05 ± 5.43	40.88 ± 6.11	0.131	.893
SAI-T1	39.30 ± 6.72	37.73 ± 5.84	1.116	.267
SAI-T2	44.65 ± 9.16	40.13 ± 8.98	2.229	.029
SAI-T3	38.43 ± 7.78	38.18 ± 7.22	0.149	.882
MoCA scores				
T1	26.18 ± 2.09	26.23 ± 1.93	−0.111	.912
T2	25.10 ± 2.51	24.66 ± 2.99	0.712	.481
T3	25.88 ± 2.02	25.34 ± 2.48	1.068	.290
ISPOCD *Z*-score				
T2	4 (10%)	6 (15%)	0.457	.261
T3	2 (5%)	5 (12.5%)	1.352	.235
VAS scores				
T1	0.25 ± 0.63	0.25 ± 0.71	0.000	.990
T2	3.23 ± 1.29	3.80 ± 1.60	−1.753	.081
T3	1.35 ± 1.25	1.53 ± 1.45	−0.595	.565

ISPOCD = International Study of Postoperative Cognitive Dysfunction, MoCA = Montreal Cognitive Assessment, SAI = State Anxiety Inventory, STAI = State-Trait Anxiety Inventory, TAI = Trait Anxiety Inventory, VAS = Visual Analog Scale.

## 4. Discussion

PND is a common operative complication in perioperative period, and its main clinical characteristics are disorder of consciousness and cognitive dysfunction, which negatively impacts the patients’ mortality, quality of life, social independence, as well as postoperative recovery and medical expenses. Neuroinflammation is one of the triggers for PND.^[17]^ Postoperative pain is also a crucial factor contributing to the occurrence of early PND. Both preoperative and postoperative pain play significant roles in the occurrence of PND.^[18]^ Zhu et al found that parecoxib can prevent early cognitive dysfunction after total knee replacement in older adults.^[19]^ Lu et al found that parecoxib preconditioning combined with dexmedetomidine can enhance early cognitive function in elderly patients following shoulder arthroscopy.^[20]^ These studies suggest that parecoxib may improve cognitive function through antiinflammatory effects. Additionally, Wang et al established a rat model demonstrating that parecoxib improves cognitive function by alleviating COX-2.^[21]^ These studies provide theoretical support for the use of parecoxib in improving postoperative anxiety and cognitive function. In our study, despite the analgesic effects of parecoxib, no significant differences in VAS scores were observed between the 2 groups, indicating that analgesia may not be the mechanism by which parecoxib alleviates anxiety and improve cognitive function. This finding may be related to the difference between analgesic and antiinflammatory mechanisms, suggesting that the antiinflammatory effects of parecoxib might be more important for alleviating anxiety and improving cognitive function.

*Z*-score is a standardized metric used to evaluate an individual cognitive function relative to the average level of a specific population. In this study, *Z*-score was used to assess cognitive function changes at different postoperative time points. Specifically, at the T2 time point (24 hours postsurgery), 6 patients (15%) in the control group had a *Z*-score that reached or exceeded a certain threshold (>1.96), while only 4 patients (10%) in the experimental group reached this threshold; at the T3 time point (7 days postsurgery), 5 patients (12.5%) in the control group reached the threshold, whereas only 2 patients (5%) in the experimental group reached it. Although these differences were not statistically significant (*P* > .05), they suggest that parecoxib may have a protective effect on cognitive function. The trend in *Z*-score indicates that the decline in cognitive function in the experimental group was relatively smaller, especially during the early postoperative stage (T2). This suggests that parecoxib may reduce the risk of postoperative cognitive dysfunction through its antiinflammatory action. For example, previous studies have shown that antiinflammatory drugs like parecoxib may reduce inflammatory responses by inhibiting COX-2, thereby protecting cognitive function.^[19]^. Furthermore, the analysis of *Z*-score reveals the trend of cognitive function changes postoperatively. Even without significant statistical differences in the short term, such trends can still provide valuable information for clinical decision-making. Similarly, it has been observed that in some cases, even if statistical significance is not achieved, the trend in cognitive function changes may indicate long-term improvements. Therefore, although the differences in *Z*-score in this study did not reach statistical significance, the trend suggests that parecoxib may have potential in reducing the risk of postoperative cognitive dysfunction. Additionally, considering the sensitivity of *Z*-score in assessing individual cognitive function, it may serve as a useful tool in clinical practice to identify high-risk patients who may experience a decline in cognitive function postoperatively.

Parecoxib is an adjunct to general anesthesia in surgery and is a selective COX-2 inhibitor used to alleviate pain, which can exert antiinflammatory effects. In this study, the SAI scores 24 hours after surgery were significantly lower in the parecoxib group compared to the control group (*P* < .05). This indicates that parecoxib may help relieve postoperative anxiety, and reducing inflammation may be one of the mechanisms responsible for this effect. However, it is noteworthy that while the SAI scores at T1 and T3 did not show significant differences between the groups, the trend towards lower SAI scores in the parecoxib group persisted throughout the observation period. This suggests that the antiinflammatory properties of parecoxib might continue to influence anxiety levels over time, potentially providing sustained benefits even if the immediate effects are not statistically significant.

Despite the lack of statistical significance in MoCA scores in this study, the results show that the cognitive function decline in the experimental group was relatively smaller. Specifically, at baseline (T1), the MoCA scores for the control group and the experimental group were 26.23 ± 1.93 and 26.18 ± 2.09 (*P* = .912), respectively, indicating that the 2 groups had comparable cognitive function levels at baseline. At 24 hours postsurgery (T2), the MoCA scores for the control group and the experimental group were 24.66 ± 2.99 and 25.10 ± 2.51 (*P* = .481), with the experimental group scoring slightly higher than the control group. At 7 days postsurgery (T3), the MoCA scores for the control group and the experimental group were 25.34 ± 2.48 and 25.88 ± 2.02 (*P* = .290), with the experimental group again scoring slightly higher than the control group. These data suggest that the experimental group experienced a smaller decline in cognitive function in the early postoperative stage (T2) and showed a slightly better recovery at 7 days postsurgery (T3). Although statistical significance was not achieved in the short term, these trends indicate that parecoxib may have a protective effect on cognitive function, particularly in reducing the risk of postoperative cognitive dysfunction. Considering the sensitivity of MoCA in assessing individual cognitive function, it may serve as a useful tool in clinical practice to identify high-risk patients who may experience a decline in cognitive function postoperatively. Future research could further validate these preliminary results by increasing sample size, extending follow-up periods, and improving evaluation tools.

There is still debate over the safety of NSAIDs and the potential risk of anastomotic leakage following intestinal tract surgery, despite enhanced recovery after surgery guidelines listing intravenous NSAIDs as the best option for minimizing opiate use after surgery.^[22,23]^ However, NSAIDs related complications such us anastomotic fistula did not occur in group P in this study. Furthermore, parecoxib did not affect the recovery of intestinal function after operation.

This study evaluated preoperative and postoperative MoCA and STAI scores, taking into account the effects of parecoxib on postoperative anxiety and cognitive performance in individuals undergoing pancreaticoduodenectomy. But there are still many limitations. Sample size and generalizability: although the sample size was calculated based on statistical power considerations, the limited number of participants (80 patients) may restrict the generalizability of the findings to broader populations. The study was conducted at a single center, which may limit the generalizability of the results to other settings or populations. Follow-up duration: the follow-up period was limited to the immediate postoperative period. Longer-term follow-up would be necessary to evaluate the sustained effects of parecoxib on cognitive function and other outcomes. Blinding: while efforts were made to blind participants, care providers, and outcome assessors, it is possible that some degree of unblinding occurred, which could introduce bias. Lack of biochemical indicators: the study did not include biochemical markers that could provide insight into the underlying mechanisms of cognitive improvement and the role of neuroinflammation. For example, markers such as interleukin-6, tumor necrosis factor-alpha, and C-reactive protein could have been measured to better understand the inflammatory response and its relationship with cognitive function. Limited evaluation metrics: the study focused primarily on the MoCA scores and SAI scores as primary outcomes. Additional cognitive tests, such as the Mini-Mental State Examination or more specialized neuropsychological tests, could have provided a more comprehensive assessment of cognitive function.

In addition, multiple outcomes and time points were analyzed without formal adjustment for multiple comparisons. This increases the possibility of type I error and may overestimate statistical significance. Therefore, the findings should be interpreted with caution, and future studies with predefined primary endpoints and correction for multiple testing are warranted.

## 5. Conclusion

This study indicates that parecoxib can alleviate postoperative anxiety, reduce the incidence of PND, and does not affect the recovery of postoperative intestinal function or increase the incidence of adverse events. Parecoxib may alleviate postoperative anxiety. Although no statistically significant reduction in PND incidence was observed, a lower occurrence was noted in the parecoxib group, indicating a possible clinical tendency that warrants further investigation.

## Acknowledgments

The authors express their appreciation to staff in The Second Hospital of Hebei Medical University, for their technical assistance.

## Author contributions

**Conceptualization:** Fuzhen Zhang, Liping Ma, Xiaoyun Li, Shizhao Wang, Lichao Di.

**Data curation:** Fuzhen Zhang, Liping Ma, Xiaoyun Li, Shizhao Wang, Lichao Di.

**Formal analysis:** Fuzhen Zhang, Liping Ma, Xiaoyun Li, Shizhao Wang, Lichao Di.

**Funding acquisition:** Fuzhen Zhang.

**Writing – original draft:** Fuzhen Zhang.

**Writing – review & editing:** Fuzhen Zhang.
